# SIRT1 Regulates Hepatocyte Programmed Cell Death via GSDME – IL18 Axis in Human and Mouse Liver Transplantation

**DOI:** 10.21203/rs.3.rs-2986981/v1

**Published:** 2023-06-27

**Authors:** Kentaro Kadono, Hidenobu Kojima, Siyuan Yao, Shoichi Kageyama, Kojiro Nakamura, Hirofumi Hirao, Takahiro Ito, Kenneth Dery, Douglas Farmer, Fady Kaldas, Xiaoling Li, Jerzy Kupiec-weglinski

**Affiliations:** David Geffen School of Medicine, University of California-Los Angeles; David Geffen School of Medicine, University of California-Los Angeles; David Geffen School of Medicine, University of California-Los Angeles; David Geffen School of Medicine, University of California-Los Angeles; David Geffen School of Medicine, University of California-Los Angeles; David Geffen School of Medicine, University of California-Los Angeles; David Geffen School of Medicine, University of California-Los Angeles; The Dumont-UCLA Transplant Center, David Geffen School of Medicine at UCLA, University of California at Los Angeles; The Dumont-UCLA Transplant Center, David Geffen School of Medicine at UCLA, University of California at Los Angeles; Signal Transduction Laboratory, National Institute of Environmental Health Sciences; David Geffen School of Medicine, University of California-Los Angeles

## Abstract

Sirtuin 1 (SIRT1) is a histone/protein deacetylase involved in cellular senescence, inflammation, and stress resistance. We previously reported that myeloid SIRT1 signaling regulates the inflamed liver’s canonical pyroptosis cell death pathway. However, whether/how hepatocyte SIRT1 is engaged in programmed cell death in the cold-stressed liver remains uncertain. Here, we undertook translational studies in human and mouse orthotopic liver transplantation (OLT) to interrogate the significance of hepatocyte-specific SIRT1 signaling in cold-stored donor livers and liver grafts after reperfusion. In the clinical arm of sixty human OLT patients, hepatic SIRT1 levels in cold-preserved donor livers correlated with anti-apoptotic Bcl-2 expression. After reperfusion, improved OLT function was accompanied by hepatic SIRT1 levels negatively associated with cleaved caspase-3 expression. In the experimental arm, we compared FLOX-control with hepatocyte-specific SIRT1-KO livers after orthotopic transplantation into WT mouse recipients, parallel with primary murine hepatocyte cultures subjected to cold activation with/without knockdown of SIRT1, GSDME, and IL18Rβ signaling. Hepatocyte SIRT1 deficiency upregulated apoptosis and GSDME-mediated programmed cell death, which in turn deteriorated the hepatocellular function and shortened OLT survival. Augmented GSDME processing, accompanied by increased secretion of IL18 by stressed hepatocytes, was prominent in SIRT1-deficient, cold-stored livers. Hepatocyte SIRT1 signaling regulated anti-apoptotic Bcl-2/XIAP proteins, suppressed cold stress-triggered apoptosis, and mitigated GSDME licensing to release IL18. Notably, while crosslinking IL18R depressed SIRT1 and Bcl-2/XIAP signaling *in vitro*, IL18 neutralization *in vivo* prevented hepatocellular damage and restored the anti-apoptotic phenotype in otherwise injury-prone SIRT1-deficient OLTs. In conclusion, this translational study identifies a novel hepatocyte SIRT1-IL18 signaling circuit as a therapeutic target in the mechanism underpinning hepatocyte death in human and mouse liver transplantation.

## INTRODUCTION

Orthotopic liver transplantation (OLT) is a life-saving therapy for end-stage liver diseases and certain hepatic malignancies (1). Despite improvements in patient survival (about 75% at 5-yrs) as a result of advances in immunosuppression and medical management, liver ischemia-reperfusion injury (IRI), an innate immune-driven response, represents a major risk factor for clinical outcomes and contributes to the shortage of organs available for transplantation. Notably, no therapeutic strategy has emerged to prevent IRI in transplant patients. Although the consequences of hepatic IRI in OLT, and potentially in other disease states involving ischemic injury/sterile inflammation, such as stroke and myocardial infarction, are vast, the mechanistic underpinnings leading to IRI are poorly understood.

The liver transplantation procedure encompasses two separate but interrelated stages of *ex vivo* cold preservation after organ procurement, and reperfusion after completing surgery in the recipient. The latter has been extensively studied in mice, but the widely-used local warm liver IRI model lacks the cold-ischemia component (2). This is a flaw because cold organ storagetriggers hepatocellular damage independently (3). Liver sinusoidal endothelial cells (LSEC) are particularly susceptible to cold stress (4), and maintaining hepatocyte integrity is essential for IRI resistance (5). Indeed, hepatocyte rather than LSEC injury markers in the liver wash-out may better predict early graft viability (6). While we have shown that stress-responsive ASK1-p38 signaling axis is involved in cold storage-triggered OLT damage (7), a complete roadmap of molecular events, particularly cell death pathways, initiated by the cold stress is needed.

Consistent with the cell death program being an essential determinant in OLT outcomes, we have reported that Bcl-2/Bcl-xL anti-apoptotic phenotype is critical for IRI resistance in murine OLT (8). However, the specific cell death cascades that operate during liver IRI remain uncertain (9). Historically, apoptosis has been considered a non-inflammatory cell death program, while necrosis a cytokine-mediated inflammatory process, and each was thought to proceed independently in a mutually exclusive manner. Recent studies, however, challenge this concept because apoptosis can crosslink with necroptosis and pyroptosis (10). Indeed, caspase-3, a key apoptosis executor, may activate Gasdermin E (GSDME), leading to a secondary programmed cell death (PCD), accompanied by the release of danger-associated molecular patterns (DAMPs), such as high-mobility group box 1 (HMGB1) and inflammatory cytokines (11, 12). Moreover, caspase-8, which mediates extrinsic apoptosis, may also control a switch between apoptosis, necroptosis, and pyroptosis (13). How these pathways communicate in IR-triggered sterile inflammation in OLT remains unknown.

To unravel molecular events initiated by liver cold preservation, we have focused on Sirtuin1 (SIRT1; silent mating type information regulation 2 homolog 1), one of the class III histone deacetylases, involved in cellular senescence, inflammation, and stress resistance. Recently, we discovered the protective function of myeloid SIRT1 signaling (14, 15) and its negative regulation of canonical inflammasome-pyroptosis in human and murine IRI-OLT (16), while others have shown that hepatocyte SIRT1 deficiency impaired lipid homeostasis/promoted steatosis in mouse livers under high-fat diet (17). As the cleavage of hepatocyte Gasdermin D (GSDMD) by caspase-1, which defines the canonical pyroptosis pathway, failed to affect liver IRI (18), while myeloid SIRT1 signaling attenuated liver IRI by suppressing caspase-1 – GSDMD activation axis (16), we attempted to study as to whether and how hepatocyte SIRT1 regulates PCD in IR-stressed OLT.

## RESULTS

### Human hepatic SIRT1 expression correlates with the apoptotic phenotype and clinical OLT outcomes

**Figure 1.**
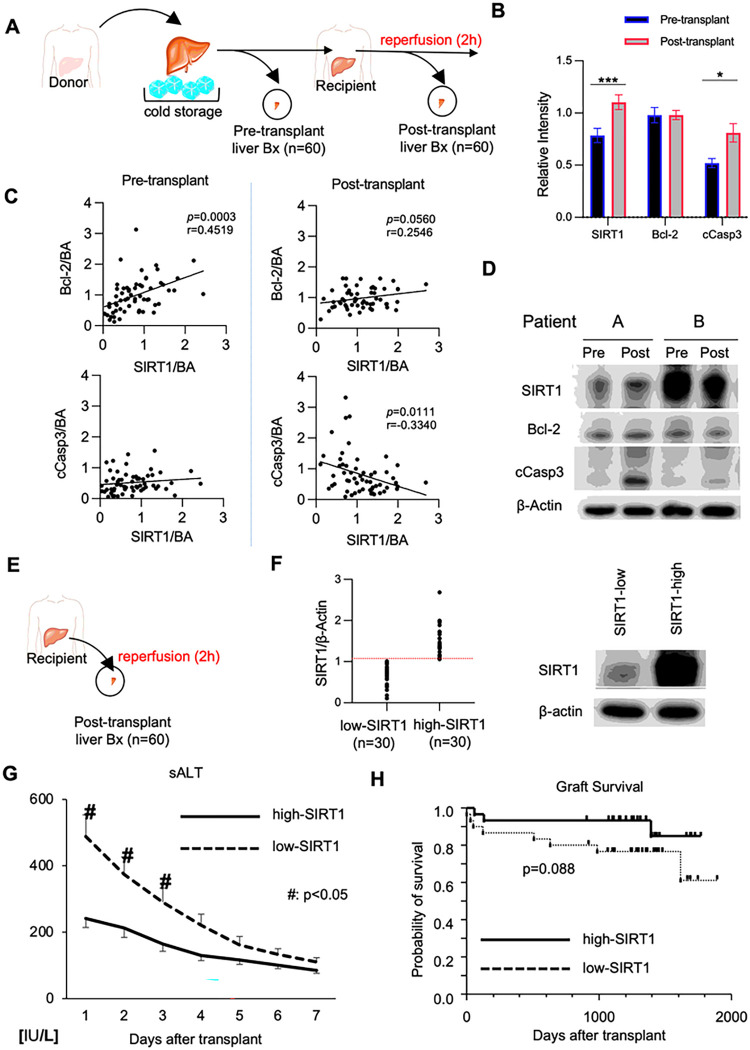
Hepatocyte SIRT1 regulates apoptosis and hepatocellular function in clinical OLT: **(A)** Human liver biopsies (Bx; n=60) were collected at the back-table (after cold-storage) and 2h post-reperfusion (before abdominal closure). **(B)** WB-assisted detection of hepatic SIRT1, Bcl-2 and cCasp3 with β-actin normalization. Data are shown in bars indicative of mean±SEM. Statistical analyses with 2-tailed Man-Whitney U test. *p<0.05; ***p<0.001. **(C)** The correlation between SIRT1 and Bcl-2/cCasp3 in pre-transplant (*left panel*) and post-transplant (*right panel*) liver Bx was analyzed by nonparametric Spearman*smethod. r*, *Spearmans* correlation coefficient. **(D)** Representative Western blots from SIRT1-low and SIRT1-high liver Bx with β-actin normalization. **(E)** Human OLT Bx samples collected 2h after reperfusion (n=60) were divided into **(F)** low (n=30) and high (n=30) SIRT1 expression groups, based on the relative SIRT1/β-actin levels. **(G)** Serum ALT levels in OLT recipients. ^#^P<0.05 (Mann-Whitney U test) **(H)** The cumulative probability of overall graft survival (Kaplan-Meier method). Solid line: high-SIRT1 group; dotted line: low-SIRT1 group (log-rank test).

We evaluated retrospectively perioperative hepatic SIRT1 expression and its correlation with hepatocellular function in a clinical cohort of sixty OLT patients. Human liver biopsies (Bx) were collected after cold storage at the back table (before transplant surgery), and post-transplant liver Bx were obtained at about two hours post-reperfusion (before abdomen closure) (Fig. 1A). Although SIRT1 signaling regulates apoptosis (19), its relationship with the apoptotic program in human OLT remains elusive. Unlike enhanced levels of cCasp3 (*P* < 0.05), anti-apoptotic Bcl-2 remained unchanged in human OLT (Fig. 1B). Indeed, SIRT1 expression correlated positively with Bcl-2 (r = 0.4519; *P* = 0.0003) in pre-transplant, and negatively with cCasp3 (r=−0.3340; *P* = 0.0111) in post-reperfusion liver Bx samples. These data indicate that SIRT1 signaling regulated apoptosis in human OLT.

To evaluate the impact of graft SIRT1 on clinical outcomes, sixty human OLTs were classified into SIRT1-low and SIRT1-high expression groups (Fig. 1F). Patients’ demographic data and clinical parameters are shown (Suppl. Table 1/2). There was no correlation between SIRT1 levels and recipient/surgical parameters, including age, gender, race, BMI, disease etiology, ABO compatibility, MELD score, pre-transplant blood tests, pre-operative hospital stay, cold/warm ischemia time, or blood transfusions during the surgery (Suppl. Table 1). There was no correlation between SIRT1 grouping and donor data (Suppl. Table 2), including age, gender, race, BMI, pre-procurement blood tests, and donation status (after circulatory or brain death).

Compared to the SIRT1-low expression group, SIRT1-high OLT patients showed improved hepatocellular function, evidenced by decreased sALT levels at post-operative days 1–3 (*P* < 0.05; Fig. 1G). To examine whether SIRT1 expression may predict clinical outcomes, we analyzed cumulative OLT survival, with median follow-up at 1280 days (range, 3–1892 days). Compared to the SIRT1-low clinical cohort, the SIRT1-high expression group displayed a noticeable trend of improved OLT survival even though it failed to reach statistical significance (Fig. 1H). This data suggests that hepatic SIRT1 expression may correlate with clinical outcomes.

### Hepatocyte SIRT1 signaling improves mouse OLT survival and suppresses apoptosis with GSDME licensing

**Figure 2.**
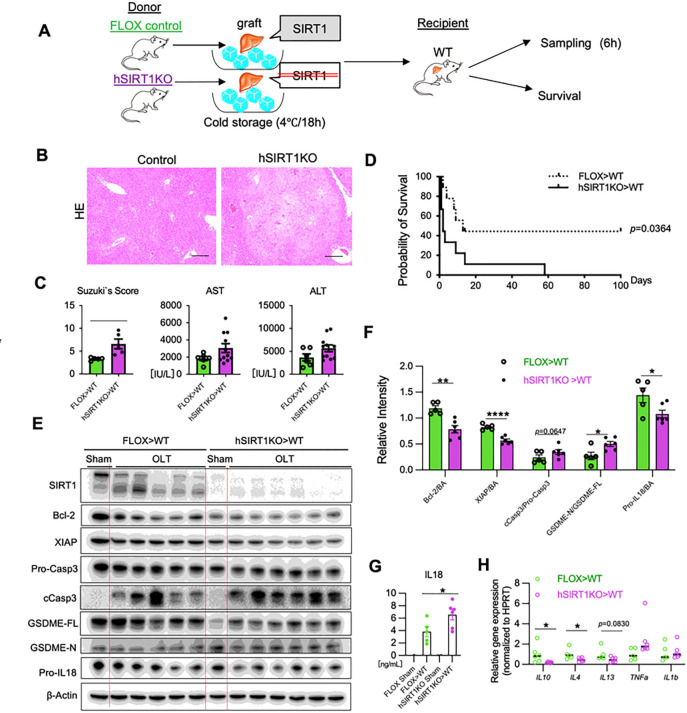
Hepatocyte SIRT1 deficiency exacerbates apoptosis and GSDME processing in mouse OLT: **(A)** Groups of FLOX control and hSIRT1KO livers, stored in UW solution (18h/4°C), were transplanted into WT mice, followed by OLT sampling at 6h. A separate OLT group was monitored for survival. **(B)** Representative OLT staining (H&E; original magnification ×100; scale bar: 200μm). **(C)** sAST, sALT levels and Suzukìs score of liver IRI. **(D)** The cumulative OLT survival (Kaplan-Meier method). Dotted line: FLOX>WT; solid line: hSIRT1KO>WT (n=9/group). **(E)** Western-assisted detection of SIRT1, Bcl-2, XIAP, Pro-Casp3, cCasp3, GSDME-FL, GSDME-N, Pro-IL18 and β-Actin in OLT at 6h. The red arrow indicates IL18. **(F)** The relative intensity ratios of Bcl-2, XIAP, cCasp3/Pro-Casp-3, GSDME-N/GSDME-FL and Pro-IL18 normalized with β-Actin. **(G)** Serum IL18 levels. **(H)** qRT-PCR-assisted detection of mRNA coding for IL10, IL4, IL13, TNFα and IL1β. Data were normalized to HPRT (n=5–6/group). Data shown are mean±SEM. *p<0.05; **p<0.01 ; ****p<0.0001 by Student`s t-test.

To gain a better understanding of how hepatocyte SIRT1 signaling may affect clinical outcomes, we applied a well-established murine model of extended *ex vivo* cold liver preservation (18h) followed by OLT, which mimics marginal human transplant setting (Fig. 2A). At 6h post-reperfusion, the peak of hepatocellular injury in this model, hSIRT1-deficient livers transplanted into WT recipients showed exacerbated sinusoidal congestion, vacuolization, and hepatocellular necrosis compared with controls (Fig. 2B/C; Suzuki’s score: hSIRT1KO > WT = 6.6 ± 1.06 vs. FLOX > WT = 3.31 ± 0.14, *P* < 0.05). These findings correlated with deteriorated hepatocellular function (sAST [IU/L]: hSIRT1KO > WT = 3062 ± 521 vs. FLOX > WT = 1843 ± 234, *P* = 0.1182 and sALT [IU/L]: hSIRT1KO > WT = 5690 ± 730 vs. FLOX > WT = 3689 ± 767, *P* = 0.1008) (Fig. 2C), and worsened OLT survival (day 100: hSIRT1KO > WT = 0% vs. FLOX > WT = 40%, *P* < 0.05 (Fig. 2D).

Having shown the association between SIRT1 and apoptotic cell death in OLT patients (Fig. 1), we next evaluated the apoptotic-pyroptotic activation profile in a murine OLT model. Indeed, the disruption of hepatocyte SIRT1 signaling decreased levels of Bcl-2 (*P* < 0.01) and X-chromosome linked inhibitor of apoptosis protein (XIAP), the caspase inhibitor (20) (*P* < 0.0001) (Fig. 2E/F). Moreover, hepatic SIRT1 deficiency increased levels of cCasp3, and the N-terminal GSDME fragment (GSDME-N) (*P* < 0.05), while decreasing Pro-IL18 (*P* < 0.05) in liver cell lysates (Fig. 2E/F). In parallel, increased levels of mature bioactive IL18 were detected in sera samples by ELISA (FLOX > WT = 3.806 ± 0.8037 vs. hSIRT1KO > WT = 6.540 ± 0.8278 ng/mL, *P* < 0.05; Fig. 2G). In the qPCR-assisted screening, disruption of hepatocyte SIRT1 signaling suppressed IL10, IL4, and IL13 while increasing TNFα levels in OLTs (Fig. 2H). These data are consistent with the idea that hepatocyte SIRT1 regulates IR-triggered liver inflammation and attenuates OLT damage by suppressing caspase-3 – GSDME-dependent PCD.

### Cold storage triggers GSDME-mediated PCD in mouse livers

**Figure 3.**
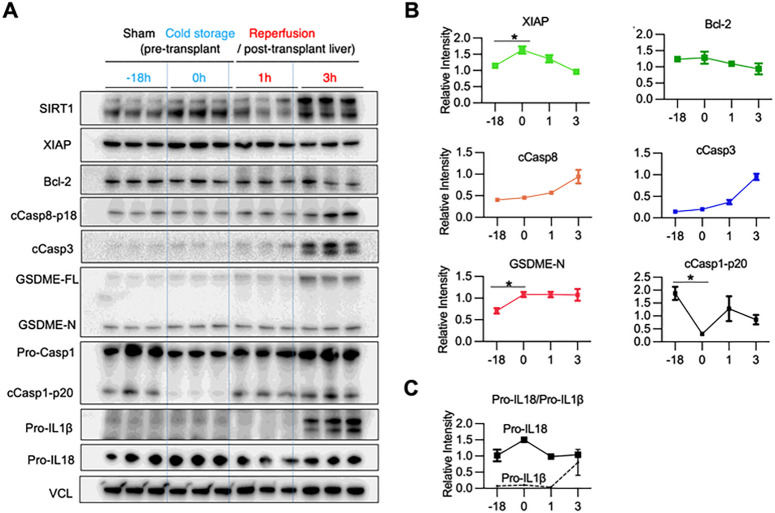
Cold preservation of mouse livers triggers GSDME processing in stressed hepatocytes: WT livers stored in UW solution (18h/4C) were transplanted into syngeneic hosts. Hepatic samples were collected after cold-storage (pre-transplant) and post-transplantation. **(A)**WB-assisted detection of SIRT1, XIAP, Bcl-2, cCasp8, cCasp3, GSDME-FL, GSDME-N, Pro-Casp-1, cCasp1-p20, Pro-IL1β and Pro-IL18 and VCL. **(B)** Kinetics of relative intensity for XIAP, Bcl-2, cCasp8, cCasp3, GSDME-N and cCasp1-p20 normalized with VCL. Data shown are mean±SEM. *p<0.05; **p<0.01 by Student`s t-test. **(C)** The relative intensity in Pro-IL18 (solid line) and Pro-IL1β (dotted line).

To determine the kinetics of apoptosis-GSDME processing, we compared cold-preserved WT livers (4°C/18h) with those after transplantation (1h and 3h post-OLT) (Fig. 3A/B). The pyroptosis executor, GSDME-N, increased after cold preservation compared to sham controls (*P* < 0.05). Although cCasp3 is a major GSDME activator, increased levels of pro-apoptotic cleaved caspase-8 (cCasp8) (*P* < 0.01), and cCasp3 were observed in transplanted livers at reperfusion, while anti-apoptotic XIAP protein significantly increased already after cold-storage. In contrast, cold stress alone markedly reduced cleaved caspase-1 (cCasp1), a marker of the canonical inflammasome-pyroptosis (Fig. 3A/B). Unlike Pro-IL1β, which increased remarkably at 3h post-OLT, Pro-IL18 was expressed throughout cold storage and reperfusion periods (Fig. 3A/C), consistent with IL18, but not IL1β, being constitutively expressed at the transcriptional and protein levels (21). Hence, IL18 becomes operational in IRI-OLT at a much earlier phase than IL1β, while GSDME-processing is already activated in cold-stressed livers before transplantation.

### Hepatocyte SIRT1 suppresses intrinsic apoptosis and GSDME-mediated PCD in cold-stored mouse livers

**Figure 4.**
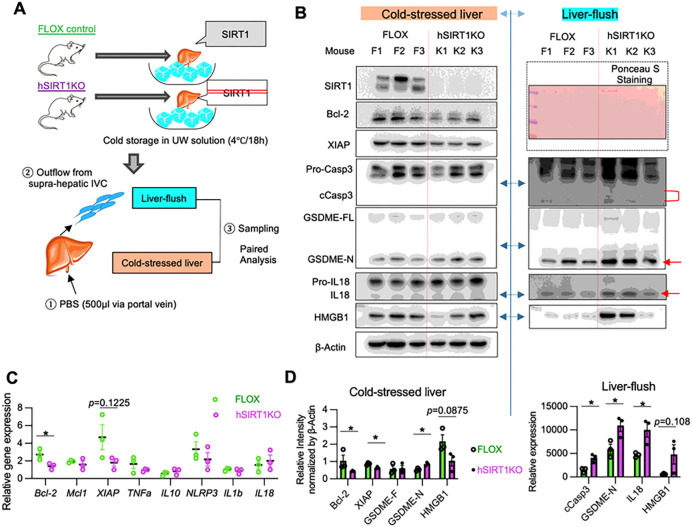
Hepatocyte SIRT1 suppresses apoptosis and GSDME-mediated PCD in cold-stored mouse and human livers: **(A)** Groups of FLOX and hSIRT1KO livers cold-stored in UW solution were perfused with physiological saline (0.5ml) via a portal vein-cuff to collect liver flush from supra-hepatic inferior vena cava (n=3/group). **(B)** WB-assisted detection of SIRT1, Bcl-2, XIAP, Pro-Casp3, cCasp3, GSDME-FL, GSDME-N, Pro-IL18, IL18, and β-actin in cold-stored livers (*left panel*). Some targets were evaluated in the liver flush by WB (*right panel*). **(C)** qRT-PCR assisted detection of mRNA coding for Bcl-2, Mcl1, XIAP, TNFα, IL10, NLRP3, IL1β and IL18. **(D)** The relative intensity of Bcl-2, XIAP, GSDME-F and GSDME-N normalized with β-actin in cold-stressed liver (*left panel*). The relative intensity of IL18, cCasp3, GSDME-N in the liver flush (*right panel*).

We next asked whether hepatocyte SIRT1 regulates apoptosis and GSDME-mediated PCD during cold liver storage. By reflecting the degree of tissue damage, liver enzymes released into the washout serve as a surrogate measure of post-reperfusion graft function (22). In addition, specific activated cell death executor proteins released into the extracellular space may be detected in the hepatic washout. Thus, we analyzed liver flush in parallel with hepatic tissue in our *ex vivo* preservation setting (Fig. 4A). Consistent with pre-transplant human Bx samples (Fig. 1C), hSIRT1-deficient cold-stressed mouse livers showed decreased gene/protein expression of Bcl-2 and XIAP (Fig. 4B/C/D) but unchanged cCasp3 levels, compared with FLOX controls (Fig. 4B). At the same time, GSDME-N trended higher in hSIRT1-KO livers, and the liver flush (*P* < 0.05) (Fig. 4B), suggesting GSDME-N may mediate PCD in cold-stored SIRT1-deficient livers. Concomitant up-regulation of cCasp3 in the liver flush confirms GSDME cleavage by cCasp3, one of its two principal proteases (11). Interestingly, we found higher levels of IL18, one of the standard mediators of GSDME activation (23), in the liver flush from SIRT1-deficient compared to FLOX livers, with little or no mature bioactive IL1β (Suppl. Figure 1). There were no changes in the canonical inflammasome activation profile between FLOX and hSIRT1-deficient cold-stressed livers, evidenced by comparable cleaved caspase-1-p20 (cCasp1-p20) and GSDMD-N levels. The ability of hepatocyte SIRT1 to promote anti-apoptotic (Bcl-2/XIAP) signaling and prevent GSDME licensing from releasing IL18 in mouse livers was confirmed in cold-stored discarded human livers (Suppl. Figure 2).

### Hepatocyte SIRT1 deficiency promotes intrinsic apoptosis and GSDME signaling in vitro

**Figure 5.**
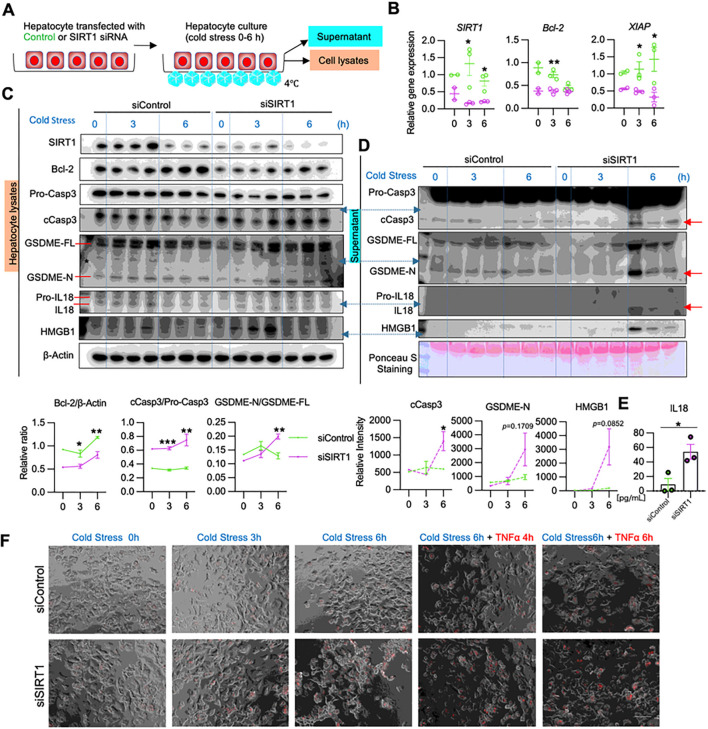
Disruption of hepatocyte SIRT1 signaling depresses the anti-apoptotic gene program and promotes GSDME processing under cold stress *in vitro*: **(A)** Primary mouse hepatocyte cultures conditioned with SIRT1 vs. control siRNA were subjected to cold stimulation. **(B)** qRT-PCR assisted detection of mRNA coding for SIRT1, Bcl-2 and XIAP. Green dots: siControl; purple dots; siSIRT1. **(C)** WB-assisted detection of SIRT1, Bcl-2, Pro-Casp3, cCasp3, GSDME-FL, GSDME-N, Pro-IL18, IL18, and β-actin in hepatocyte lysates (*upper panels*). The relative intensity ratios of Bcl-2, cCasp3/Pro-Casp3, GSDME-N/GSDME-FL (*lower panels*). Green line: siControl; purple line: siSIRT1. **(D)**WB-assisted detection of Pro-Casp3, cCasp3. GSDME-FL, GSDME-N, Pro-IL18 and IL18 in the culture medium (*upper panels*). Ponceau S staining is shown as a loading control. The relative intensity ratios of cCasp3 and GSDME-N (*lower panels*). Green line: siControl; purple line: siSIRT1. **(E)** ELISA-assisted IL18 levels in cold-stressed hepatocytes. Data shown are mean±SEM. *p<0.05; **p<0.01; ***p<0.001 by Student`s t-test. **(F)** Composite images of phase contrast and immunofluorescence propidium iodide staining of primary mouse hepatocytes conditioned with control (*upper panels*) or SIRT1 (*lower panels*) siRNAs under cold stress (3, 6h), followed by TNFα stimulation (4, 6h; 25ng/mL). Original magnification ×200; scale bar; 100μM.

To further elucidate how SIRT1 regulates hepatocyte apoptosis and GSDME-mediated PCD, we screened for the expression of apoptotic and pyroptotic markers in primary mouse hepatocyte cultures, with/without siRNA-silencing of SIRT1 upon cold stimulation (Fig. 5A). SIRT1-deficient hepatocytes showed suppressed Bcl-2/XIAP gene expression in vivo (Fig. 5B), while SIRT1-silencing down-regulated hepatocyte Bcl-2 protein but upregulated cCasp3/GSDME levels *in vitro* (Fig. 5C). Consistent with cold-stored livers (Fig. 4), SIRT1-silenced hepatocyte cultures secreted significant amounts of IL18 (*P* < 0.05; Fig. 5D/E), concomitantly with cleaved forms of cCasp3/GSDME (Fig. 5D). These data imply that SIRT1 signaling promotes anti-apoptotic Bcl-2 and XIAP proteins to prevent cold stress-induced cell death cascade. Notably, although hepatocytes have never been reported to secrete IL18, we observed cold-stressed SIRT1-deficient hepatocytes to release ample IL18 (Fig. 5D/E).

Whether hepatocytes can undergo pyroptosis regardless of gasdermin activation remains controversial (24). Although in our study, SIRT1-silenced hepatocytes displayed up-regulated cell membrane permeability, evidenced by increased propidium iodide (PI) uptake, we observed cell shrinkage rather than swelling or rupture, cardinal morphologic features of hepatocyte pyroptosis (Fig. 5F). To mimic the OLT reperfusion phase, we then supplemented cold-stressed hepatocyte cultures with a TNFα adjunct. Indeed, unlike in controls, the addition of TNFα readily triggered PCD in SIRT1-silenced hepatocytes (Fig. 5F).

### SIRT1 regulates cold stress-induced apoptosis and GSDME licensing to release IL18

**Figure 6.**
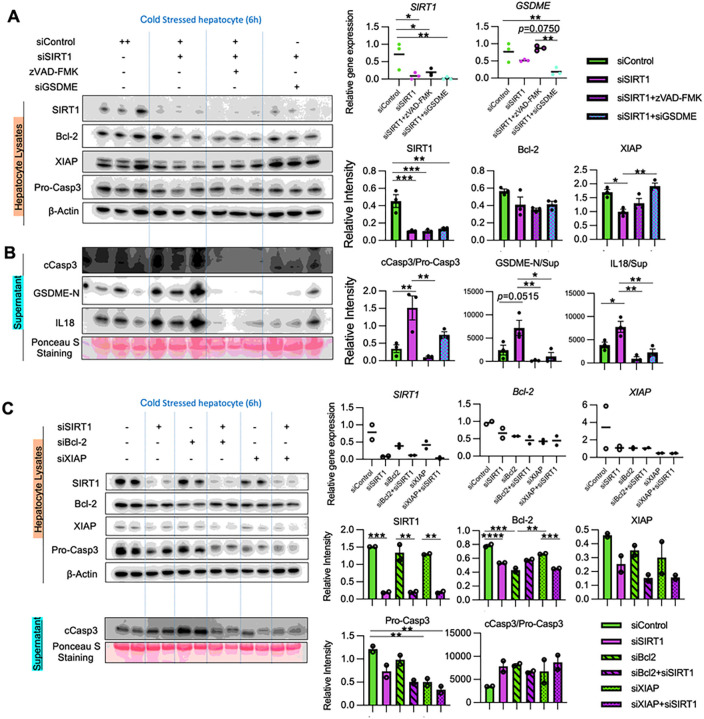
SIRT1 regulates cold stress-induced apoptosis signaling and GSDME processing to release IL18 *in vitro*: **(A-B)** Primary mouse hepatocytes pretreated with control or SIRT1 siRNA were subjected to cold stress. Some SIRT1-silenced cells were pretreated with a pan-caspase inhibitor zVAD-FMK (20nM, 18h) or transfected with GSDME siRNA. **(A)** WB-assisted detection of SIRT1, Bcl-2, XIAP, Pro-Casp3 and β-actin in lysates (*left panels*). qRT-PCR-assisted detection of mRNA coding for SIRT1 and GSDME (*right upper panels*). The relative intensity of SIRT1, Bcl-2 and XIAP in lysates (*right lower panels*). **(B)**cCasp3, GSDME-N and IL18 in supernatants (*left panels*). The relative intensity of cCasp3/Pro-Casp3 ratio, GSDME-N and IL18 in supernatants (*right panels*). **(C)** Primary mouse hepatocytes pretreated with SIRT1 or/and Bcl2 or/and XIAP siRNA were subjected to cold stress. WB-assisted detection of SIRT1, Bcl-2, XIAP, Pro-Casp3 and β-Actin in hepatocyte lysates (*left upper panels*) and cCasp3 in supernatants (*left lower panels*). The relative intensity of SIRT1, Bcl-2, XIAP and cCasp3/Pro-Casp3 ratio (*right panels*). Data shown are mean±SEM. *p<0.05; **p<0.01; ***p<0.001;****p<0.0001 by one-way ANOVA.

We used a siRNA approach to address the role of GSDME in hepatocyte PCD under cold stress. Indeed, GSDME-silenced hepatocyte cultures showed remarkable suppression of IL18 and HMGB1 secretion (Suppl. Figure 3). Furthermore, to elucidate the relationship between SIRT1, apoptosis/GSDME-mediated PCD, and IL18 release, we treated SIRT1-silenced hepatocytes with zVAD-FMK, a pan-caspase inhibitor, or transfected SIRT1-silenced hepatocytes with GSDME-siRNA, before cold stimulation (Fig. 6A/B). First, adjunctive conditioning with zVAD-FMK significantly suppressed hepatocyte GSDME activation, compared to SIRT1-silenced cells alone, indicating GSDME activation under cold stress was caspase-dependent. Second, IL18 release in SIRT1-silenced hepatocytes was reduced after treatment with zVAD-FMK or transfection with GSDME-siRNA. These data suggest that hepatocyte SIRT1 regulates IL18 release in a caspase- and GSDME-dependent manner. Notably, GSDME-silenced hepatocytes displayed increased levels of XIAP compared with SIRT1 silencing alone. This suggests that anti-apoptotic proteins control cell death and PCD may affect the anti-apoptotic protein program.

Next, we investigated whether Bcl-2/XIAP are essential for SIRT1 regulation of caspase activation. With silencing Bcl-2 or XIAP, SIRT1-knock downed hepatocytes failed to augment caspase 3 activation compared to controls (Fig. 6C). Thus, Bcl-2/XIAP are critical for SIRT1 regulation of apoptosis signaling in cold-stressed hepatocytes.

### IL18 signaling suppresses hepatocyte SIRT1 and the anti-apoptotic protein axis

**Figure 7.**
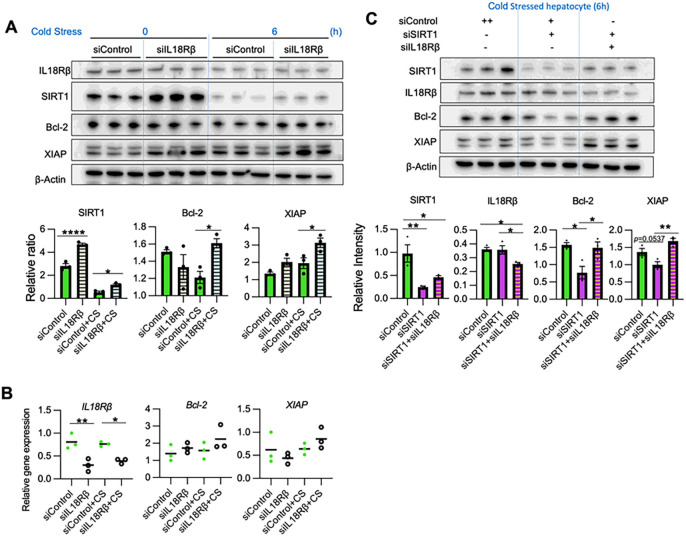
IL18 signaling suppresses hepatocyte SIRT1 and anti-apoptotic axis: Primary hepatocytes pretreated with control or IL18Rβ siRNA were subjected to cold stress. **(A)** WB-assisted detection of IL18Rβ, SIRT1, Bcl-2, XIAP and β-actin (*upper panels*). The relative ratio of SIRT1, Bcl-2 and XIAP (*lower panels*). **(B)**qRT-PCR-assisted detection of mRNA coding for IL18Rβ, Bcl-2 and XIAP. **(C)** Primary hepatocytes conditioned with control or SIRT1 and/or IL18Rβ siRNA were subjected to cold stress. WB assisted detection of SIRT1, IL18Rβ, Bcl-2, XIAP and β-Actin (*upper panels*). The relative intensity of SIRT1, IL18Rβ, Bcl-2 and XIAP (*lower panels*). Data shown are mean±SEM. *p<0.05; **p<0.01; ****p<0.0001 by one-way ANOVA.

To investigate the effect of IL18 on hepatocytes, we silenced the IL18 receptor β unit (IL18Rβ), which is specific for hepatocyte IL18 response and indispensable for high-affinity IL18 binding (25). Interestingly, IL18Rβ knockdown up-regulated SIRT1, Bcl-2, and XIAP under cold stress and without IL18 stimulation (*P* < 0.05) (Fig. 7A/B), suggesting that extracellular IL18 can accelerate the hepatocyte death. Then, to evaluate whether IL18R regulation of anti-apoptotic factors is SIRT1-dependent, we knock-downed IL18Rβ in SIRT1-silenced hepatocytes. While SIRT1-silencing alone depressed hepatic Bcl-2/XIAP levels, concomitant transfection with IL18Rβ siRNA rescued Bcl-2/XIAP in SIRT1-silenced hepatocytes (Fig. 7C), suggesting IL18R-mediated Bcl-2/XIAP regulation is SIRT1-independent. Thus, IL18R is critical for SIRT1 regulatng anti-apoptotic programs in cold-stressed hepatocytes.

### IL18 neutralization mitigates liver damage and restores anti-apoptotic phenotype in OLT

**Figure 8.**
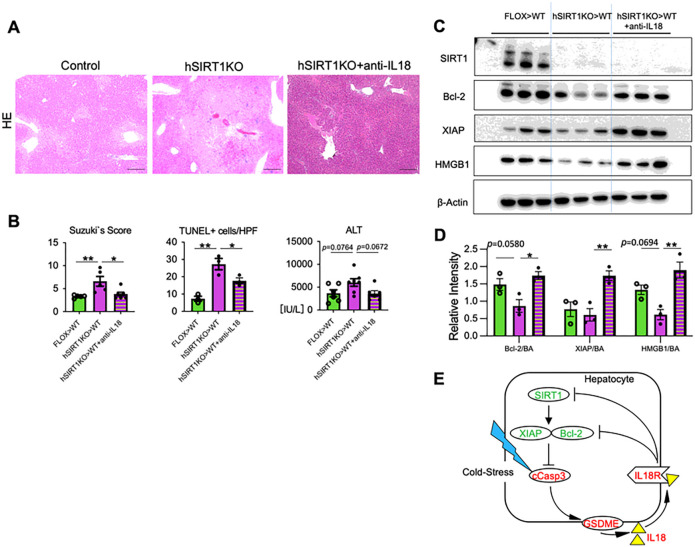
IL18 neutralization prevents liver IR damage and promotes anti-apoptotic phenotype in SIRT1-deficient murine OLT: Groups of FLOX control and hSIRT1KO livers, stored in UW solution (18h/4°C), were transplanted into WT mice that remained untreated or pretreated with anti-IL18 Ab (0.5mg i.p. at −1 day), followed by sampling at 6h. **(A)** Representative H&E staining (original magnification ×100; scale bar 200μm); **(B)** Suzukìs score of liver IRI; frequency of TUNEL+ cells/HPF (original magnification ×200; scale bar 100μm); and sALT levels. **(C)** Western-assisted detection of SIRT1, XIAP, Bcl-2, HMGB1 and β-actin in OLTs. **(D)** The relative intensity ratios of XIAP, Bcl-2 and HMGB1 normalized with β-actin. Data shown are for n=5–7/group (sALT/Suzuki’s score) and n=3/group (TUNEL staining/WB); mean±SEM. *p<0.05; **p<0.01 by one-way ANOVA. **(E)** Schematic illustration of proposed cold stress-triggered hepatocyte cell death programs. SIRT1 regulates the activation of caspase3, followed by GSDME activation to release IL18 via the Bcl-2/XIAP anti-apoptotic axis. The secreted IL18 further down-regulates SIRT1, Bcl-2 and XIAP.

As IL18R – IL18 axis was critical for homeostatic SIRT1 regulation of anti-apoptotic programs in cold-stressed hepatocytes *in vitro*, we aimed to assess *in vivo* function of IL18 in IRI-OLT. Groups of hSIRT1KO (cold-stored) livers were transplanted to WT recipients with/without adjunctive anti-IL18 mAb conditioning. As shown in Fig. 8A/B, by 6h post-reperfusion, IL18-neutralized OLTs showed well-preserved histological detail, with decreased sinusoidal congestion, vacuolization, and hepatocellular necrosis (Suzuki’s score: hSIRT1KO > WT + anti-IL18 = 3.83 ± 0.47 vs. hSIRT1KO > WT = 6.6 ± 1.06, *P* < 0.05), diminished frequency of TUNEL + cells/HPF (hSIRT1KO > WT + anti-IL18 = 17.67 ± 1.76 vs. hSIRT1KO > WT = 27.33 ± 3.28, *P* < 0.05), and improved function (sALT [IU/L]: hSIRT1KO > WT + anti-IL18 = 3616 ± 628 vs. hSIRT1KO > WT = 6043 ± 835, *P* = 0.067). Moreover, consistent with siIL18Rβ-silenced SIRT1-deficient hepatocyte cultures (Fig. 7C), disruption of IL18 signaling *in vivo* restored the anti-apoptotic phenotype in hSIRT1KO liver grafts, consistent with increased levels of Bcl-2/XIAP, and decreased HMGB1 release, indicating that liver grafts were less vulnerable to IRI in IL18-deficient environment (Fig. 8C/D).

## DISCUSSION

This translational study uncovered the regulatory function of hSIRT1 in the mechanism of cold stress-induced hepatocyte death in human and mouse OLT. Clinical liver transplant screening revealed that SIRT1 signaling correlated positively with the anti-apoptotic cell death phenotype and negatively with the extrinsic apoptotic pathway. The experimental arm established a new hepatocyte cell death feedback loop in which GSDME licensed extracellular IL18 to depress the inflamed liver’s homeostatic SIRT1 signaling and anti-apoptotic phenotype.

We have reported that myeloid-specific SIRT1 regulates the canonical inflammasome assembly in IR-stressed liver (16). Having confirmed the decrease of Pro-IL18 in IRI-OLT and increased IL18 levels in serum and the liver flush under cold stimulation (Fig. 2H/4B), we expected SIRT1 to exert a similar regulatory function in the stressed hepatocytes. However, with no difference in caspase-1/GSDMD signaling between FLOX-control and hSIRT1-deficient livers (Suppl. Figure 1/4), we noticed increased apoptosis and GSDME-mediated PCD selectively in hSIRT1KO livers. Notably, unlike hepatocyte SIRT1 deficiency, disruption of myeloid SIRT1 did not affect apoptosis in IR-stressed mouse liver (Suppl. Figure 5). To the best of our knowledge, this is the first report to document the GSDME-mediated PCD pathway in hepatic IRI-OLT.

GSDME of the gasdermin family initiates pyroptosis following cleavage by caspase-3 (11). It is also essential for releasing DAMPS, such as HMGB1 (12, 23), and inflammatory cytokines, such as IL1β, IL18 (26), and IL1α (27). Here, hSIRT1-deficient cold-stored livers and SIRT1-silenced hepatocytes were enriched in IL18 but not mature bioactive IL1β. Moreover, siRNA-facilitated silencing confirmed that hepatocyte GSDME regulated IL18 release under cold stress. In contrast to IL1β, which requires amplifying the NF-κB precursor for activation, IL18 is constitutively expressed in unstimulated cells (28). Therefore, detecting hepatocyte-derived IL18 rather than IL1β was not surprising, although no previous reports show stressed hepatocytes to release IL18. Indeed, IL18 has been primarily associated with activating NK/Th1 cells and IFN-γ production in response to intracellular infection (29). Although IL18 induces extrinsic apoptosis in hepatocytes (30) and endothelial cells (31), GSDME-silenced hepatocytes showed increased XIAP with decreased IL18 levels in our study, indicating that GSDME-licensed IL18 depressed the anti-apoptotic protein program. Rogers et al. reported that GSDME augmented apoptosis in lymphoid cell lines/macrophages by forming mitochondrial pores independently of Bid truncation, a pro-apoptotic protein of the Bcl-2 family (23). As GSDME processing down-regulated Bcl-2/XIAP to activate caspase-3, IL18Rβ-silenced hepatocytes had significantly increased anti-apoptotic protein levels (Fig. 7A/B). Mouse hSIRT1-deficient recipients showed higher IL18 serum and lower hepatic IL18 levels, with augmented apoptosis and GSDME processing. Bysani et al. demonstrated that endothelial cell-derived IL18 might induce inflammation/apoptosis in myocardial cells via autocrine and paracrine signaling (31). Hence, we have identified a novel mechanism by which GSDME-licensed IL18 bridges the apoptosis feedback loop in cold-stressed hepatocytes. Although further studies are needed to elucidate how IL18 can augment hepatocyte apoptosis, our data are consistent with the idea that hepatocyte-derived IL18 should be regarded as a new player in the pathogenesis of inflammatory liver diseases. Strikingly, while crosslinking IL18R depressed SIRT1 and Bcl-2/XIAP signaling *in vitro*, neutralization of IL18 *in vivo* exerted beneficial biological function by mitigating IR-triggered hepatocellular damage and restoring the anti-apoptotic phenotype in otherwise injury-prone SIRT1-deficient OLTs (Fig. 8).

Although cCasp3 activates GSDME-mediated PCD, a recent study showed that granzyme B could process GSDME in cytotoxic CD8 T lymphocytes (32). While disruption of hSIRT1 signaling in the donor liver (KO) augmented cCasp3, the kinetic analysis showed apoptosis occurred primarily after reperfusion in SIRT1-proficient livers (WT), accompanied by up-regulation of XIAP during cold stress (Fig. 3). In agreement with augmented caspase3/GSDME activation, disruption of SIRT1 signaling suppressed anti-apoptotic phenotype both *in vivo* and *in vitro* (Fig. 4/5). These data imply that an anti-apoptotic program in the donor liver is essential for hepatocyte resistance against IR-stress. Similarly, Megan et al. reported that the inactivation of Mcl-1/Bcl-xL in keratinocytes during viral infection promoted GSDME-dependent pyroptosis to release IL1α intrinsically (27). Thus, our results are consistent with the idea that SIRT1 – Bcl-2/XIAP cross-talk safeguards against cold stress response and IR-triggered sterile inflammation in the liver.

Consistent with our murine data, hSIRT1 signaling was associated positively with Bcl-2 in human OLTs (Fig. 1), confirming the clinical relevance of SIRT1-mediated cytoprotection. The investigation into the extracellular GSDME-N – IL18 feedback loop revealed that apoptosis and GSDME-mediated PCD became operational during cold activation, both *in vivo* and *in vitro*. Recently, machine perfusion has been shown to improve the quality of marginal human liver grafts and extend the preservation time, enabling therapeutic modulation/perfusate biomarker monitoring (33). Hence, the SIRT1 – Bcl-2/XIAP signaling axis might provide new targets for decreasing hepatocyte damage in liver transplant patients while disrupting the GSDME-N – IL18 axis may promote “rejuvenation” of marginal livers during perfusion preservation.

In summary, hepatocyte SIRT1 – Bcl-2/XIAP signaling negatively regulated the apoptosis feedback loop, bridged by GSDME-licensed extracellular IL18 in IR-stressed OLTs (Fig. 8E). This pathway highlights a novel mechanism in hepatocyte PCD, and provides putative therapeutic targets against acute/chronic inflammatory liver diseases.

## MATERIALS/SUBJECTS AND Methods

### Clinical liver transplant study

Human studies were approved by the UCLA Institutional Research Board (IRB 13-000143), and written informed consent was received from participants. We performed a retrospective analysis of sixty adult patients who underwent OLT (May 2013-August 2015) and received routine standard of care and immunosuppressive therapy. Recipients who underwent re-transplantation were excluded from the study. Donor livers, procured from donation after brain or cardiac death, were perfused/stored in the UW solution (Niaspan; Bristol-Meyers Squibb). Protocol Tru-Cut needle Bx were obtained from the left lobe after cold storage (at the back table) about 2h after portal reperfusion (before abdominal closure) and snap-frozen. Hepatic Bx were screened by Western blots with β-actin normalization for SIRT1, Bcl-2, and cCasp3 expression. Recipient blood samples were evaluated for sALT levels.

### Animals

C57BL/6 male, wild-type (WT; Jackson Laboratory, Bar Harbor, ME), FLOX, and hepatocyte SIRT1-deficient (hSIRT1-KO; NIEHS, Research Triangle Park, NC) mice were used. Animals were housed under pathogen-free conditions, and received care outlined in the Guide for Care and Use of Laboratory Animals (National Academies Press, 2011).

### Mouse model of orthotopic liver transplantation

All mouse experiments were approved by the UCLA Animal Research Committee (ARC 1999–094). We used our a mouse model of *ex vivo* hepatic cold storage and OLT (8). To mimic marginal human transplantation setting and focus on hepatic SIRT1 while avoiding host alloimmune responses, donor livers (FLOX or hSIRT1-KO; BL6) stored in UW solution (18h/4°C) were transplanted to syngeneic recipients. In some experiments, prospective WT recipients of hSIRT1KO liver grafts were conditioned with a neutralizing anti-IL18 Ab (0.5mg i.p. at 24h before surgery; YIGI74-1G7; Bio-x-Cell). Liver graft/serum samples were collected at 6h post-reperfusion. To investigate the influence of cold ischemia alone, hepatic tissue/liver flush samples (after infusion of 0.5ml saline via a portal vein cuff) were collected after cold preservation.

### Hepatocyte isolation and cultures

Primary mouse hepatocytes, isolated by a 2-stage collagenase perfusion method (34), were cultured (3h/6h) with/without cold stress (4°C). Hepatocytes were silenced with siRNA targeting SIRT1, GSDME, Bcl-2, XIAP and IL18Rβ (Santa Cruz Biochemistry) using Lipofectamine (Invitrogen) before cold stimulation. In some experiments, hepatocytes were pre-treated with a pan-caspase inhibitor (zVAD-FMK; R&D Systems).

### Hepatocyte cell death screening

Cultured hepatocytes were incubated with propidium iodide (PI; 1μg/ml for 5min) and screened with a fluorescence microscope for phase-contrast images.

### Statistics

For human data, continuous values were analyzed by Mann-Whitney *U* test and categorical variables by Fisher*sexacttest*. *Spearmans* correlation coefficient (r) was used to evaluate the strength of linear relationship between variables. For mouse data, comparisons between two or multiple groups were assessed using a Student *st* − *test* and *o* ≠ − *wayanalysisofvariance*(*ANOVA*), *followedbyTukeys* HSD (honest significance difference) test. All *P* values were 2-tailed and *P* < 0.05 was considered statistically significant. Analyses were performed with GraphPad Prism software.

## Data Availability

All data generated during this study are available from the corresponding author upon reasonable request.
